# Efficient gene delivery and selective transduction of astrocytes in the mammalian brain using viral vectors

**DOI:** 10.3389/fncel.2013.00106

**Published:** 2013-07-05

**Authors:** Nicolas Merienne, Juliette Le Douce, Emilie Faivre, Nicole Déglon, Gilles Bonvento

**Affiliations:** ^1^Laboratory of Cellular and Molecular Neurotherapies, Department of Clinical Neurosciences, Lausanne University HospitalLausanne, Switzerland; ^2^Commissariat à l’Energie Atomique et aux Energies Alternatives, Département des Sciences du Vivant, Institut d’Imagerie Biomédicale, Molecular Imaging Research Center and CNRS CEA URA 2210Fontenay-aux-Roses, France

**Keywords:** viral vectors, astrocytes, CNS, tropism, gene therapy

## Abstract

Astrocytes are now considered as key players in brain information processing because of their newly discovered roles in synapse formation and plasticity, energy metabolism and blood flow regulation. However, our understanding of astrocyte function is still fragmented compared to other brain cell types. A better appreciation of the biology of astrocytes requires the development of tools to generate animal models in which astrocyte-specific proteins and pathways can be manipulated. In addition, it is becoming increasingly evident that astrocytes are also important players in many neurological disorders. Targeted modulation of protein expression in astrocytes would be critical for the development of new therapeutic strategies. Gene transfer is valuable to target a subpopulation of cells and explore their function in experimental models. In particular, viral-mediated gene transfer provides a rapid, highly flexible and cost-effective, *in vivo* paradigm to study the impact of genes of interest during central nervous system development or in adult animals. We will review the different strategies that led to the recent development of efficient viral vectors that can be successfully used to selectively transduce astrocytes in the mammalian brain.

Astrocytes make up most of the cells in the brain. In addition to well-characterized roles for astrocytes in regulating brain metabolism and blood flow, there is now an increasing body of evidence that astrocytes are dynamic regulators of synaptogenesis, synaptic function and network activity. This is conceptualized in the tripartite synapse model, where pre-synaptic and post-synaptic elements of neurons are surrounded and regulated by astrocyte processes (Araque et al., 1999; Barres, 2008).

Astrogenesis occurs relatively late in development after most neurogenesis has completed (Freeman, 2010). Defects in astrocyte maturation, tripartite synapse formation and plasticity during early post-natal development may be responsible for some psychiatric and neurodegenerative diseases. There is a growing body of evidence to support the view that a loss of normal astrocyte functions or a gain of abnormal effects can contribute to disease processes, and there are now numerous examples of astrocyte contributions to pathological mechanisms in amyotrophic lateral sclerosis (ALS), Huntington’s disease (HD), and brain tumors to cite few of them (for review see Sofroniew and Vinters, 2010).

Despite progress and potential significance, cellular, developmental, and systems-level studies of astrocytes still lag far behind those of neurons. New sophisticated genetic tools to label and manipulate astrocytes *in vivo *were recently developed. Additional tools that allow for temporally controlled deletion of genes, specifically in rodent astrocytes, along with improved high resolution imaging techniques, are enabling researchers to address fundamental questions in astrocyte biology for the first time. However, these tools need to be more fully expanded and exploited to better understand astrocyte biology *in vivo*. The situation is complicated by the recent findings that astrocytes do not represent a homogeneous cell population across brain regions as well as within the same brain region (Zhang and Barres, 2010). So, despite evidence showing pronounced region- and layer-specific morphological heterogeneity as well as region-specific actions of astrocytes on neuronal functions, currently available tools have had limited utility for examining functional diversity among astrocytes.

To understand the role of astrocyte signaling in brain function, it is critical to study astrocytes *in situ* where their complex morphology and intimate association with neurons remains intact. Understanding neuron–glia interactions *in vivo* requires dedicated experimental approaches to manipulate each cell type independently. These approaches include targeted transgenesis and viral transduction to overexpress or block the expression of a specific gene in astrocytes.

The past and current approaches of targeted transgenesis were recently reviewed in a comprehensive paper (Pfrieger and Slezak, 2012) and will not be detailed here.

Yet, a very important application of transgene expression is the visualization of a large population of astrocytes *in vivo* by a fluorescent protein. The use of bacterial artificial chromosomes (BACs) for the production of transgenic mice has opened new opportunities to study gene expression and functions in the brain. The resulting gene expression central nervous system (CNS) atlas program GENSAT represents a powerful resource for the scientific community (http://www.gensat.org). However, it remains difficult and time-consuming to target specific cell subpopulations through transgenesis, and differences in recombination efficiency between transgenic lines complicate the analysis. We will therefore rather focus on an alternative approach to genetically manipulate astrocytes that relies on the use of viral vectors. Indeed, the development of highly efficient viral vectors for gene transfer in the CNS is providing new systems for localized and controlled gene expression. Even if such approach requires the stereotaxic injection of the viral vectors in each animal, it significantly reduces the costs of *in vivo* experiments, and it can be used in combination with mouse models for conditional gene targeting, providing high flexibility and versatility to replace, modify, induce, or block expression of target genes. We will therefore review the recent development in this field that led to the emergence of effective and selective viral vectors for transducing astrocytes *in vivo*.

## VIRAL VECTORS: POTENT SYSTEM FOR *IN VIVO* GENE DELIVERY IN BRAIN

**FIGURE 1:**
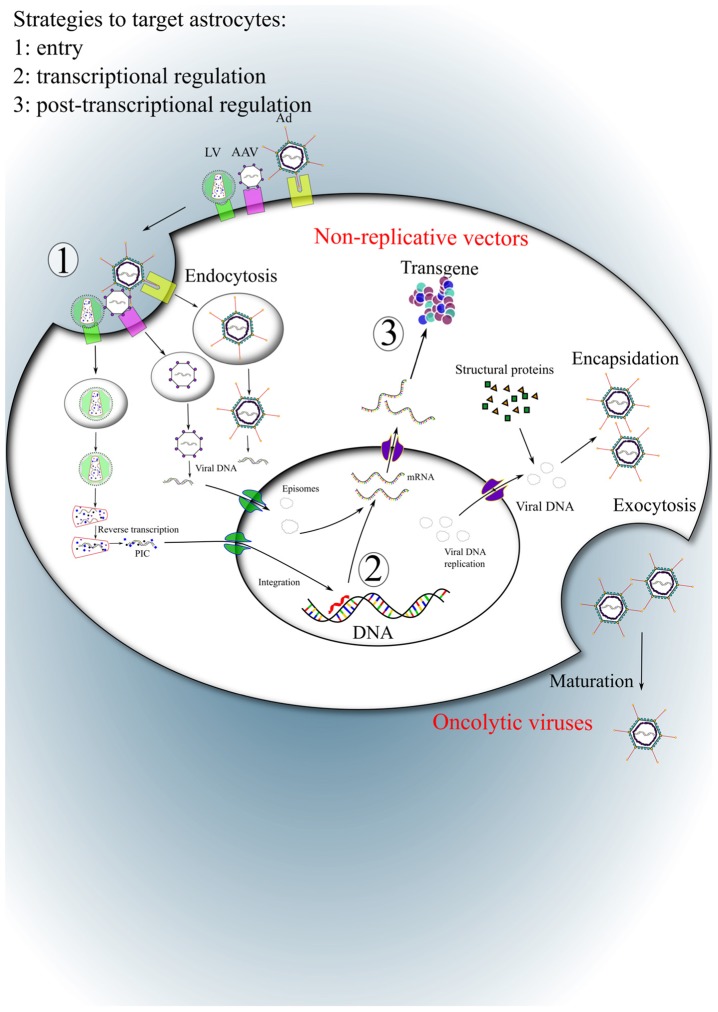
**Strategies to target astrocytes.** Three steps of viral cycle are used to modify the tropism of viral vectors: (1) the entry, (2) the transcriptional and (3) post-transcriptional regulations. After binding to their respective receptors, LV, AAV, and Ad enter into host cells via receptor-mediated endocytosis. Viral DNA (AAV and Ad) or RNA (LV) are uncoated in the cytoplasm. The viral DNA remains as extrachromosomal episomes in the nucleus while viral RNA is integrated into the host genome after reverse transcription. For non-replicative vectors, in most cases only the transgene is expressed. In the case of oncolytic viruses, viral genes encoding structural proteins are necessary for the encapsidation and production of replicative particles. PIC, pre-integration complex.

Viral vectors offer the possibility to control expression of a transgene in adult or developing brain areas and can exploit the unique ability of viruses to deliver genetic material into mammalian cells. Viral vectors are derived from various viruses and are engineered to preserve the transduction efficiency while preventing the original pathogenicity and, in most cases, the capacity to multiply (Davidson and Breakefield, 2003). These viral vectors are often called multiply attenuated and replication-deficient viral vectors (**Figure 1**). Among the most widely used vectors for CNS applications are the lentiviral (LVs) and adeno-associated viral vectors (AAVs) which have particularly attractive properties which include, the capacity to infect non-dividing cells, the absence of cytotoxic or immune response, long-term transgene expression and large diffusion in the brain. At least for LV, the cloning capacity is sufficient to integrate most of the genes of interest (Déglon and Hantraye, 2005). Viral vectors provide a gene transfer tool that is independent of age and species considered (Kay et al., 2001; Kirik et al., 2003; Lundberg et al., 2008). Along with somatic gene transfer in developing or adult animals, viral vectors can also be used for transgenesis in species in which classical methods are not suitable, in particular large animals (Yang et al., 2008; Wongsrikeao et al., 2011).

Natural viruses have a specific pattern of infection, which reflects the recognition and interaction between viral capsid/envelope and receptors expressed on susceptible cells. Similarly, the tropism of viral vectors is primarily determined by the interaction of the viral surface proteins with receptor molecules expressed on target cells but other mechanisms could be used for subpopulation-restricted gene transfer in the brain. In particular, cell-type-specific promoters, post-transcriptional regulatory elements, replacement of retroviral envelope proteins with heterologous viral surface proteins, a phenomenon called pseudotyping (Page et al., 1990) or the use of various serotypes (AAV and Ad harboring different capsids) have been proposed to dissect and elucidate gene functions in astrocytes.

The first viral vector was obtained by exploiting the natural tropism of brain cells from the Herpes simplex virus type 1 (HSV-1; Geller and Breakefield, 1988; Federoff et al., 1992). The HSV-1 genome is complex and large, but replication-incompetent vectors, with a partial (first generation of HSV-1 vectors) or complete (amplicons) deletion of viral genes allow the insertion of very large transgenes (around 150 kb). The HSV-1 amplicons are neither pathogenic nor toxic for the infected cells and are retrogradely transported to the CNS from the peripheral nervous system (PNS; Frampton et al., 2005). These vectors have a widespread tropism for neurons (Jerusalinsky et al., 2012) and similarly to AAV and adenoviral vectors, their genetic material does not integrate into the host genome thus reducing the risk of insertional mutagenesis (Manservigi et al., 2010). However, HSV amplicons are difficult to produce, elicit low levels of adaptive immune responses and most of the human population is seropositive which limits their clinical applications for chronic disorders (Manservigi et al., 2010).

A few years after the apparition of HSV vectors, adenoviral vectors (Ad) were derived from the Ad type 5 serotype (Le Gal La Salle et al., 1993; Horellou et al., 1994). These vectors also have a high cloning capacity (approximately 30 kb of double-stranded DNA for gutless Ad) but the tropism of these vectors is not naturally oriented to the brain (Arnberg, 2012). Interestingly, a live (replication-competent Ad) vaccine has been safely administered to humans (Rubin and Rorke, 1994). This vaccine program reflects the strong immune response induced by Ad in humans (White et al., 2011), a reason why these vectors are promising candidates for tumoral therapy, and are proposed for the treatment of glioblastoma (Candolfi et al., 2006; Kroeger et al., 2010).

In the mid 1990s, the first AAV (from serotype 2) and LVs were reported (Page et al., 1990; Kaplitt et al., 1994; Naldini et al., 1996). The AAV vectors are derived from the smallest non-enveloped viruses (approximately 20 nm) and have a cloning capacity of 5 kb of single-stranded DNA. The AAV2 naturally infects humans but is non-pathogenic. It is classified as a dependovirus because it requires a co-infection with a helper virus such as Ad or HSV to perform its infectious replication cycle. The AAV persists for years in transduced cells mostly as an extrachromosomal episome (Nakai et al., 2001; Schnepp et al., 2005). To date, more than 100 serotypes of AAV have been identified, each of them possessing a specific tropism in the CNS due to the binding of the capsid with specific receptors (Wu et al., 2006a b). Fourteen clinical trials using AAV gene transfer were performed to assess their potential therapeutic value in various neurodegenerative diseases (Crystal et al., 2004; Tuszynski et al., 2005; Kaplitt et al., 2007). In 2012, the first AAV gene therapy product was marketed by the European Medicine Agency (EMEA) for the treatment of patients suffering from lipoprotein lipase deficiency (Yla-Herttuala, 2012).

Finally, the most extensively characterized LVs are derived from HIV-1, which is a subclass of retroviruses. Retroviruses are lipid-enveloped particles comprising a homodimer of linear, positive-sense, single-stranded RNA genomes of 7–11 kb. Following entry into target cells, the RNA genome is retro-transcribed into linear double-stranded DNA and integrated into the cell chromatin (Delelis et al., 2010). To decrease the risk of insertional mutagenesis, integration-deficient LVs (IDLV) were designed (Wanisch and Yanez-Munoz, 2009). These IDLVs are based on the use of integrase mutations that specifically prevent proviral integration, a process that results in the generation of increased levels of circular vector episomes in transduced cells. LVs were tested clinically for the treatment of adrenoleukodystrophy (ALD) and Parkinson’s disease (PD). In the case of ALD, an *ex vivo* approach was used, with the transduction of hematopoietic CD34+ cells and re-infusion of corrected cells in the patients. An immunological improvement occurred in the two treated children aged 9–12 months in combination with a blockage of the demyelinating lesions observed by magnetic resonance imaging (MRI), 12–16 months after gene therapy (Cartier et al., 2009 2012). In a second study, a dopamine replacement strategy, with an LV that encodes the three enzymes responsible for the production of dopamine was tested in a phase I/II clinical trial. Increasing doses of LV were injected into the striatum of 15 patients with mid-stage PD. An improvement in motor function was observed at 6-months relative to pre-treatment assessment (Palfi, 2008; Jarraya et al., 2009 and see (http://www.oxfordbiomedica.co.uk).

## STRATEGIES TO TARGET ASTROCYTES

The understanding of astrocyte functions in normal and altered brain strongly relies on the availability of experimental systems to specifically target astrocytes *in vivo*. However, the first generation CNS viral vectors had a strong neurotropism *in vivo *(Naldini et al., 1996; Hermens and Verhaagen, 1997; Rabinowitz and Samulski, 1998). Indeed, the injection of AAV2 into adult rodent brains was associated with neuronal transgene expression when using ubiquitous promoters (Bartlett et al., 1998; Mandel et al., 1999; Bjorklund et al., 2000). Similarly, stereotaxic injection into rat or mouse brain of LVs pseudotyped with the vesicular stomatitis virus glycoprotein (VSV-G) with CMV (cytomegalovirus) or PGK (phosphoglycerate kinase 1) promoters, leads to the specific transduction of neurons with very limited transgene expression in other cell types (Naldini et al., 1996; Kordower et al., 1999; Déglon et al., 2000). Finally, the Ad5 displays a partial neurotropism with the transduction of other cell types, especially astrocytes (Smith et al., 1996; Bohn et al., 1999; Soudais et al., 2001; Rubio and Martin-Clemente, 2002; Wang et al., 2012).

**FIGURE 2:**
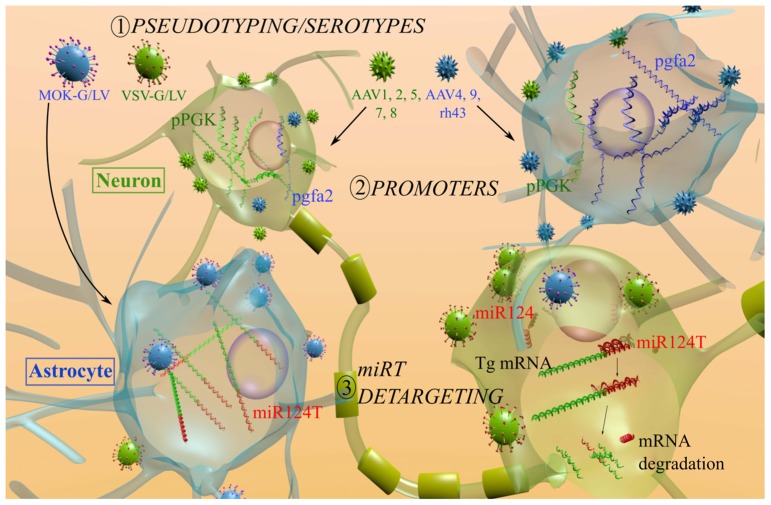
**Mechanisms used to restrain the transgene expression of AAV and LV in astrocytes.** (1) To modify the entry, various AAV serotypes or LV pseudotyping with heterologous VSV-G (green) and MOK-G (blue) envelopes were used. The tropism of LV is mainly neuronal (green cells) with the VSV-G envelope and a partial shift toward astrocytes (blue cells) is observed with the MOK-G envelope. AAV1, 2, 5, 7, and 8 mainly transduce neurons (green) while AAV4, 9, rh43 display a partial astrocytic tropism. (2) To restrict transgene expression, astrocytic promoters were investigated (cells in the upper part). Transgene expression under the control of a PGK promoter (pPGK, green mRNA) leads to a preferential expression in neurons, whereas a gfa2 promoter (pgfa2, blue mRNA) results in an astrocytic expression. (3) To block the transgene expression in unwanted cells (lower part), miRNA target (miRT) sequences are integrated in the 3′-UTR of the vector (red signal on the green mRNA). The miR124 is exclusively expressed in neurons. As a consequence the miR124T is only recognized in neurons and the transgene expression is blocked (mRNA degraded). miR124, microRNA 124; miR124T, miR-124 target sequence; Tg. transgene.

However, it is important to mention that a number of parameters could alter the tropism. These include, amongst other factors, the purity of the vector, the mode of production, the site of administration, species, the developmental stage, and normal or pathophysiological conditions. Unfortunately, data gathered in primary cultures (neurons and astrocytes) are not predictive of the *in vivo* tropism and a systematic evaluation of each vector is still required. Indeed, VSV-G/LV-GFP under the control of various promoters efficiently transduces primary rat astrocytes and to a lesser extent mouse astrocytes (Englund et al., 2000; Li et al., 2010) while transgene expression is mainly restricted to neurons *in vivo *(Naldini et al., 1996; Kordower et al., 1999; Déglon et al., 2000). This phenomenon was also observed with AAV2, which efficiently targets astrocytes *in vitro* but not *in vivo *(Gong et al., 2004). The purification method has also a major impact on the tropism of AAV8. In the mouse hippocampus, the CsCl-purified AAV8-CMV-GFP displayed an astroglial pattern in contrast to the expected neuronal expression obtained with an iodixanol purification method (Klein et al., 2008). Foust et al. (2009) found that injection of AAV9-CMV early enhancer/chicken β actin promoter (CAG)-GFP into the tail vein of adult mice mainly transduces astrocytes throughout the CNS (Foust et al., 2009), whereas the tropism is mainly neuronal after intracerebral injection or intravenous injection in neonatal mice (Klein et al., 2008). Finally, discrepancies have been observed on the transduction efficiency and tropism of various AAV serotypes between species (rodent, cat, and primates; Davidson et al., 2000; Vite et al., 2003; Burger et al., 2004; Gray et al., 2011). Additional studies are therefore still warranted to fully characterize the tropism of these vectors in the CNS. However, three strategies to direct viral vectors toward astrocytes have already been developed: shifting the tropism by favoring the entry of viruses in astrocytes, limiting transgene expression with astrocytes-specific promoters or blocking transgene expression in unwanted cells (**Figure 2**).

### ALTERING THE ENTRY OF VIRAL VECTORS

The tropism of a virus is first determined by its binding with a specific receptor at the surface of the host cell (Lutschg et al., 2011; Arnberg, 2012). Knowledge of the structure and viral capsids or envelopes and their corresponding receptors provide essential information to specifically target individual cell types and/or diseased tissues. For example, the tropism of Ad5 vectors is regulated by the binding to its primary cellular receptor; the coxsackie and adenoviral vectors receptor (CAR). Tissues refractory to Ad5 infection do not express CAR. The limited expression of CAR in dopaminergic neurons of the substantia nigra of mice explains the poor transduction of these cells and transgene expression in astrocytes and other non-neuronal cells (Lewis et al., 2010). However, the expression of CAR in the nervous system and in particular in glial cells has not been extensively examined and CAR-independent forms of Ad have been developed to shift the tropism (Grellier et al., 2011).

As mentioned above, more than 100 serotypes of Ad and AAV were characterized but only a dozen of them infect cells of the CNS. Indeed, for most of them, only limited data are available concerning their receptors and their pattern of expression in the brain. The earliest and most used serotype is the AAV2, which has a natural tropism for neurons (Bartlett et al., 1998; Kugler et al., 2003). The binding of AAV2 to its primary receptor, the heparan sulfate proteoglycan (HSPG) has been well-characterized, and is centered around two amino acids on the spikes of the AAV2 capsid (Kern et al., 2003; Opie et al., 2003). However, HSPG is necessary, but not wholly sufficient, for the transduction of permissive cells. In addition, fibroblast growth factor receptor 1 (FGFR-1) was identified as a co-receptor of AAV2 (Qing et al., 1999). The tropism of AAV5 *in vivo* correlated with the pattern of expression of platelet-derived growth factor receptor (PDGFR)-alpha (Di Pasquale et al., 2003). The AAV1, 5, 7, 8, and 9 not only infect astrocytes *in vivo* but also neurons and other cells (Davidson et al., 2000; Wang et al., 2003; Shevtsova et al., 2005; Cearley and Wolfe, 2006; Gray et al., 2011). The AAV9 is unique compared to other AAV serotypes in that it is capable of crossing the blood–brain barrier and transducing neurons and/or astrocytes in the brain depending of the developmental stage (Foust et al., 2009). Recently, it has been shown that AAV9 uses galactose at the N-linked glycans as a receptor (Bell et al., 2011; Shen et al., 2011). The identification of the amino acids of the AAV9 capsid necessary for binding to galactose opens the possibility to modify the tropism (Bell et al., 2012). Finally, AAV4 and AAVrh43 preferentially target astrocytes (Liu et al., 2005; Lawlor et al., 2009) but the receptors for these serotypes are unknown. AAV4-RSV-βGal and AAVrh43-CAG-eGFP exclusively transduce astrocytes when injected into the subventricular zone (SVZ) or the striatum. However, AAVrh43-CAG-eGFP infects approximately 3 mm^3^ of the striatum and 2,000 astrocytes per mm^3^ while AAV8-CAG-eGFP infects 6 mm^3^ of the striatum and 150,000 neurons per mm^3^ (Lawlor et al., 2009).

Lentiviral vectors are increasingly being used in neuroscience research and are unique in the sense that they are enveloped viruses that can be pseudotyped (i.e., the original envelope protein can be replaced by heterologous glycoproteins). The most used pseudotype for LV is VSV-G which confers some interesting properties to the vector (**Figure 2**). It dramatically broadens LV tropism by facilitating transduction of various cell types in different species, it stabilizes the vector particles from shear forces during centrifugation thereby allowing vector concentration and it directs LV to an endocytic pathway, which reduces the requirements of viral accessory proteins for transduction (Cockrell and Kafri, 2007). Initial studies suggest that VSV-G/LV enters into cells using phosphatidylserine (PS), but there is no correlation between the cell surface PS levels and VSV infection or binding (Coil and Miller, 2004). In addition, competition for PS using antagonists does not block the binding of VSV on target cells. Currently, the receptors responsible for VSV-G/LV entry in cells are unknown.

In the CNS, VSV-G/LVs expressing transgenes under the control of ubiquitous promoters have mainly a neuronal tropism with a limited transgene expression in astrocytes (Naldini et al., 1996; Déglon et al., 2000; Watson et al., 2002). Among the other envelopes used to pseudotype LVs, lymphocytic choriomeningitis virus (LCMV) and Mokola virus (MOK) envelopes result in a partial transduction of astrocytes. *In vivo*, LV/LCMV infects specifically astrocytes in the substantia nigra and in the striatum (Miletic et al., 2004; Cannon et al., 2011). Injection of MOK/LV into the striatum or the hippocampus leads to the infection of cells that are mainly astrocytes (Pertusa et al., 2008; Colin et al., 2009). Although no quantifications were done using LCMV/LV, 70% of cells transduced by MOK/LV are astrocytes, 20% are neurons and 10% are other cell types of the striatum. In addition, it is important to note that the titers and the transduction efficiency of these latter vectors are usually lower than VSV-G/LV.

In conclusion, specific serotypes or envelopes only partially improve the astrocytic targeting of viral vectors. However, engineering chimeric capsids or envelopes targeting astrocytes is difficult and time-consuming. In order to optimize viral vectors tropism, strategies aiming at restraining transgene expression with astrocytic promoters, or by blocking expression in unwanted cells, mainly in neurons, were developed.

### TARGETING ASTROCYTES WITH TRANSCRIPTION REGULATORY ELEMENTS

Different astrocytic promoters have been used to restrict transgene expression into glial cells. However, the packaging size of each viral vector limits the type of promoters which can be inserted. Analysis of the transcriptional regulatory elements of the glial fibrillary acidic protein (GFAP) promoter reveals that 5′-flanking regions of the gene are sufficient to direct transgene expression in astrocytes (Brenner et al., 1994). Two fragments compatible with AAV and LV vectors were created: gfa2 of 2.2 kb and gfaABC1D of 600 bp (Brenner et al., 1994; Lee et al., 2008). The cloning of the gfa2 fragment into Ad5 and AAVrh43 vectors restricts transgene expression in rat astrocytes (Do Thi et al., 2004; Lawlor et al., 2009; Mamber et al., 2010; Arregui et al., 2011). However, no quantification was performed to determine the number of transduced astrocytes. In the study by Lawlor et al. (2009), the gfa2 promoter was cloned into the AAV8 vector. The Gfa2-AAV8 vector infects mainly astrocytes in the striatum but a low transgene expression was still observed in neurons. The authors indicated that AAV8-gfa2-eGFP has high transduction efficiency with a wide diffusion in the striatum while AAVrh43-gfa2-eGFP transduces only a limited number of cells. It was shown recently that injection of high titer of AAV5-gfa2-eGFP into the striatum or the substantia nigra provides an astrocyte-specific expression with no residual expression into neurons or microglial cells. In addition, the expression was stable until 12 weeks post-injection. Stereological analysis of transgene expression reveals that a mean of 15,000 astrocytes per mm^3^ of striatal tissue were transduced (Drinkut et al., 2012), corresponding to ~75% of the astrocytes present in the transduced area (Savchenko et al., 2000).

Astrocytic promoters were also used in combination with LCMV and MOK pseudotyped LVs (**Figure 2**). The vector LCMV/LV-gfa2-Cre was injected into the SVZ of Rosa26 mice that express the sequence LoxP-stop-LoxP-LacZ (Stein et al., 2005). The expression of Cre in transduced cells removes the STOP cassette in Rosa26 mice and as a consequence, LacZ staining was observed in astrocytes of the SVZ after LCMV/LV injection. However, no quantification was performed although some neurons expressed the transgene. To develop an expression system activated in pathological conditions, Jakobsson et al. (2004) took advantage of GFAP up-regulation in reactive astrocytes. Using toxin-induced lesion models (6-hydroxy-dopamine and ibotenic acid lesions), they showed that the transgene expression is eightfold higher in reactive astrocytes: a finding which correlates with the activity of the endogenous GFAP gene (Jakobsson et al., 2004). Recently, other astrocytic promoters were used in LV vectors, such as the glutamate transporter promoter, EAAT1 (Colin et al., 2009). In this study, striatal injection of MOK/LV-EAAT1-GFP leads to the expression of the transgene mainly in astrocytes (75% of the transduced cells).

In conclusion, astrocyte-specific promoters alone or in combination with an “astrocytic” capsids or envelopes, significantly shift the tropism of viral vectors toward astrocytes *in vivo*. However, the targeting is, in most cases, not complete and a residual transduction (10–40%) of non-astrocytic cells is observed. In addition, most studies rely on the use of the GFAP promoter. Large initiatives are underway to characterize the regulatory elements of the whole human genome (Gerstein et al., 2012; Whitfield et al., 2012) and new astrocyte-specific promoters were recently described. For example, the aldehyde dehydrogenase 1 family, member L1 (ALDH1L1) promoter is highly active in all mature astrocytes (Cahoy et al., 2008) while the GLAST promoter was used to express transgene in GFAP-positive but also GFAP-negative astrocytes (Liu et al., 2006; Regan et al., 2007; Buffo et al., 2008). Analysis of GLAST and GLT1-GFP mice has revealed an unexpected non-overlapping pattern between the two transporters and confirmed the differential activation of the promoters during embryogenesis and in adulthood. GLAST activity was low in the forebrain and high in the cerebellum, whereas GLT1 expression was higher in the cortex than in the cerebellum, consistent with the prominent role of GLT-1 in glutamate uptake in the forebrain. Combining data from the ENCODE project and the gene expression cartography in human and mouse brain will provide additional and essential information to identify minimal fragments necessary for cell-type-specific transgene expression in viral vectors (Hawrylycz et al., 2012). This strategy has already been developed by the Pleiade Project, which integrated information from genomic databases to construct synthetic MiniPromoters for viral vectors containing only the indispensable regulatory elements to achieve gene expression (Portales-Casamar et al., 2010).

### DETARGETING STRATEGY USING MICRORNA

To further improve viral vector tropism, post-transcriptional regulatory elements have been integrated into viral vectors to block transgene expression in non-targeted cells. This strategy called “detargeting” uses microRNA (miRNA) machinery to obtain tissue-specific expression (Brown et al., 2007; **Figure 2**). miRNAs are small non-coding RNA of 19–25 nucleotides that mediates post-transcriptional gene suppression (Bartel and Chen, 2004; O’Carroll and Schaefer, 2013). Approximately 1,000 miRNAs have been identified and almost 50% of them are expressed in mammalian brains (He et al., 2012). These miRNA are differentially distributed in distinct brain regions and show cell-type specificity with even differential intraneuronal miRNA compartmentalization (Bak et al., 2008; Edbauer et al., 2010). Since miRNAs target most genes, they represent important regulators of expression and are implicated in a large range of biological activities. The negative regulation of gene expression is mediated through base-pairing with complementary regions within the 3′ untranslated region (3′-UTR) of their target protein-coding messenger RNAs (mRNAs; Bartel and Chen, 2004; Kosik, 2006; Saugstad, 2010). To restrict transgene expression in a specific cell population, a miRNA present in unwanted cells but not expressed in targeted cells is chosen. A natural target sequence (miRT) or a sequence fully complementary to the mature miRNA is cloned in the 3′-UTR of the gene of interest (Brown et al., 2007). This detargeting strategy was first demonstrated in the CNS with the neuron-specific miR124 (Colin et al., 2009). In this latter study, four copies of the natural target sequence of miR124 from the integrin-β1 gene were inserted in a LV to block transgene expression in neurons. When a miRT with a partial complementarity (bulged miRT) to its miRNA is placed in 3′-UTR of a gene of interest, repression occurs both at post-transcriptional (mRNA degradation) and translational levels. Whereas, in the case of a synthetic miRT with full complementarity with the miRNA, mRNA degradation is the main mechanism of action (Gentner and Naldini, 2012). Importantly, no saturation of miRNA machinery or adverse biological effects was reported with these miRNA-regulated LV (Colin et al., 2009; Gentner et al., 2009). The miRT threshold for saturation varies for each miRNA, perfectly complementary miRTs have a lower risk to saturate the miRNA machinery. In addition, each miRNA has differential suppressive activity ranging from 5 up to >150-fold (Gentner et al., 2009). In this context, miR124 is a promising candidate because it is highly expressed in neurons (Lagos-Quintana et al., 2002; Smirnova et al., 2005; Deo et al., 2006). The insertion of four miR124T sequence in a VSV-G pseudotyped LV (VSV/LV-PGK-LacZ-miR124T) significantly decreases transgene expression levels and the number of β-galactosidase-positive neurons in the striatum of adult mice (Colin et al., 2009). This detargeting approach was used to shift the tropism of LV toward astrocytes. Double-immunofluorescence staining with neuronal and astrocytic markers demonstrated that combining mokola pseudotyping and miR124T (MOK/LV-PGK-LacZ-miR124T) resulted in a transgene expression that was almost exclusively restricted to astrocytes, with 89 ± 3% β-galactosidase-S100β-positive cells and 6 ± 4% NeuN-positive cells. This effect was not restricted to the striatum as similar results were obtained in the hippocampus and cerebellum.

**FIGURE 3:**
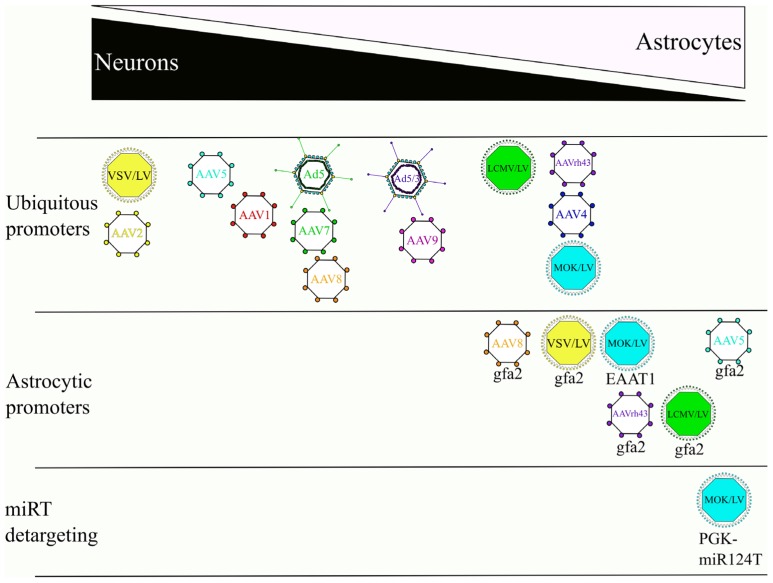
**Effects of the envelope/serotype, promoter, and miRT detargeting on the cellular tropism of LV, AAV and Ad.** Overview depicting the tropisms of viral vectors in the CNS. References used for this figure are detailed and cited in the text.

In conclusion, the use of these three different strategies (modulation of viral vector entry, transcription and post-transcriptional regulations) has enabled the development of efficient gene transfer systems to specifically target astrocytes (**Figure 3**). Thanks to the unique features of these new viral vectors, it has already been possible to make significant advances in two areas of research related to the development of innovative therapies and the modeling of neurological disorders.

## VIRAL VECTORS TARGETING ASTROCYTES: APPLICATIONS FOR BRAIN DISEASES

### MODELING BRAIN DISEASES

There is evidence to support the idea that the mechanisms responsible for selective neurodegeneration in some brain disorders are non-cell autonomous and based upon pathological cell–cell interactions. The selective death of the neuronal population at risk in each disorder can be better explained by the convergence of multiple pathogenic mechanisms which provoke damage within the vulnerable neuron and neighboring cell types rather than by autonomous cell mechanisms (Ilieva et al., 2009).

In order to dissect out the specific role of different cell populations *in vivo* (neurons, astrocytes, microglia), two different strategies were recently used. The first one relies on the use of the Cre/loxP system to silence the expression of the mutant protein in specific cell types by crossing different Cre-expressing transgenic mice with transgenic mice expressing the mutant protein flanked by loxP sites in all cell types. The opposite strategy consists of selectively expressing the mutant protein in specific cell types using either specific promoters such as GFAP or by crossing different Cre-expressing transgenic mice with transgenic mice expressing the mutant protein after a STOP cassette flanked by loxP sites.

These two strategies were useful in providing evidence that astrocytes play a key role in the pathogenesis of ALS (Ilieva et al., 2009), spinocerebellar ataxia 7 (Custer et al., 2006), HD (Gu et al., 2005; Bradford et al., 2009 2010), and taupathies (Forman et al., 2005; Dabir et al., 2006). However, an alternative strategy based upon the use of viral vectors to selectively and locally express the mutant protein has also proven to be very useful and complementary to the development of transgenic mice in particular to test whether a local expression is sufficient to induce pathological mechanisms. Through the use of a newly developed LV (Colin et al., 2009), a short form of the mutant protein huntingtin (mHtt, responsible for HD), was expressed only in striatal astrocytes and not in neurons (Faideau et al., 2010). It has been shown that these glial cells developed a progressive phenotype of reactive astrocytes that was characterized by a marked decreased expression of both glutamate transporters, GLAST and GLT-1, and of glutamate uptake. This reactive phenotype was associated with neuronal dysfunction, as observed by a reduction in DARPP-32 and NR2B expression. Consistent with the above findings, a histological re-evaluation of potential astrocyte reactivity within postmortem brains of HD patients showed the presence of astrogliosis in the caudate nucleus of Grade 0 patients and confirmed the colocalization of mHtt in astrocytes with a grade-dependent reduction in GLT-1. Through the use of viral vectors that target astrocytes locally, we were able to show that the presence of mHtt in astrocytes is sufficient to alter the glial glutamate transport capacity early in the disease process and may contribute to pathogenesis of HD.

### GLIOBLASTOMA MULTIFORM

Glioblastoma multiform (GBM) is the most common primary tumor developing in the brain from astrocytes. Due to the quick proliferation and its infiltrative nature, complete ablation by surgery is almost impossible. The prognosis is very poor, with a median survival of 14.6–19.6 months and an inevitable relapse within a few months after the resection (Grossman et al., 2010). Viral-mediated gene therapy aiming to reduce glial proliferation represents, therefore, an alternative therapy (Murphy and Rabkin, 2013). Indeed, GBM is a good candidate for gene therapy because tumor cells rarely develop metastasis outside of the brain and most cells in the CNS are post-mitotic, reducing side effects of therapeutic strategies targeting dividing cells.

However, appropriate viral vectors for the treatment of GBM are different from those developed for the treatment of neurodegenerative diseases. For GBM therapy, the aim is to mediate destruction of proliferating cells. Glial targeting is achieved either by the injection of the vector into the tumor mass, by choosing a vector which target dividing cells or having a partial tropism for glial cells, as it is the case for Ad (Asadi-Moghaddam and Chiocca, 2009).

The first studies used a replication-deficient mouse moloney leukemia virus (MLV) that infected dividing cells and expressed a suicide gene (thymidine kinase, TK; Ram et al., 1993). Thymidine kinase is a phosphotransferase enzyme that incorporates dGTP analogs in the presence of ganciclovir instead of cellular dGTP and leads to the blockade of cellular replication (Boivin et al., 1993). But the low transduction efficiency neither improved tumor progression nor the overall survival time (Ram et al., 1993 1994; Gunzburg et al., 1995). To improve the efficacy of the treatment, vector-producing cells (VPC releasing MLV particles expressing the TK suicide gene) were injected into the brain after surgical resection of the tumor. However, no significant decrease of tumor mass occurred despite the bystander effect (Ram et al., 1997; Klatzmann et al., 1998; Shand et al., 1999; Packer et al., 2000; Rainov, 2000; Martinet et al., 2003). As an alternative therapy, Ad-TK was administered directly to GBM patients but the phase III trial showed no positive outcome (Cottin et al., 2011). Interestingly, it was shown that the preferential transduction of glioma cells is not dependent on the expression of known Ad receptors on tumor cells (Candolfi et al., 2006). Expressing the therapeutic suicide gene under the control of a strong ubiquitous promoter in combination with an immune stimulator may increase therapeutic efficacy and prevent relapse (Candolfi et al., 2006; Ghulam Muhammad et al., 2009).

As an alternative strategy to improve the therapeutic efficacy, conditionally replicative or replicative viruses were developed. The principle of oncolytic therapy is to inject directly into the tumoral cells a lytic replicative-competent cytotoxic virus, such as HSV, VSV, Ad, or retroviruses, which will induce apoptosis in proliferative cells during replication (Parker et al., 2009; Zemp et al., 2010; Castro et al., 2011; Russell et al., 2012). HSV were initially used as lytic viruses in GBM therapy (Zemp et al., 2010). However, the high worldwide HSV seropositivity limits their use in the clinic and as a consequence has led to the development of other oncolytic viruses. A deletion of E1B region on Ad genome (Ad-ONYX-15) was introduced to favor apoptosis in infected glioma cells but the efficiency of this approach was too low to reach a phase II of clinical trial (Moran, 1993; Chiocca et al., 2004). In addition, replicative adenoviral vectors expressing therapeutic genes were used to mediate tumoral cells destruction. The candidate genes are inserted in the E3 deleted region and a CAR-independent entry mechanism enhancing the transduction efficiency of tumoral cells has been proposed for these new generation oncolytic viruses. To favor replication in GFAP-positive cells, three copies of glial specific B enhancer were added on the gfa2 promoter (gfa2B3), leading to a decreased growth of glioma cells (Horst et al., 2007).

### GENE THERAPY FOR NEURODEGENERATIVE DISORDERS

Degeneration of the nigro-striatal projection represents the major pathological hallmark of PD. Preclinical rodent and non-human primate models demonstrated a strong protective effect of glial cell line-derived neurotrophic factor (GDNF) on the nigro-striatal dopaminergic system (Gash et al., 1996; Kirik et al., 2000). However, intrathecal infusion of GDNF protein or viral vector-mediated expression of neurturin in the striatum of late stage PD patients showed no significant clinical benefit (Lang et al., 2006; Marks et al., 2010). Current gene therapeutic trials in the brain predominantly use AAV2 due to its proven safety record. In the animal and human CNS, AAV2 predominately transduces neurons. However, the expression of neurotrophic factors in neurons may impose a serious safety issue since the factors can be secreted from the soma, unmyelinated projections, or synaptic sites of transduced neurons, thereby delivering a complex signaling-inducing molecule to potential off-target sites. One alternative strategy would be to restrict their impact to the immediate vicinity of the site of the lesion. Through the use of an AAV5 expressing GDNF under the expression of GFAP, Drinkut et al. (2012) demonstrated the same efficacy as neuron-derived GDNF. In terms of safety, unilateral striatal GDNF expression in astrocytes did not result in delivery of bio-active GDNF to the contralateral hemispheres (potential off-target sites) as was the case when GDNF was expressed in neurons. This suggests that astrocytic neurotrophic factor expression achieved by a viral vector can be considered an efficient alternative to current gene therapeutic strategies.

Astrocyte activation, characterized by hypertrophic somata and processes, is an early hallmark in most neurodegenerative conditions. The functional impact of this activation on the progression of these diseases is still elusive and their therapeutic potential is yet unexploited. A recent study has taken advantage of the strong astrocytic tropism of AAV2/5 expressing the astrocyte-specific promoter Gfa2 to test the potential of astrocyte-targeted therapeutics in an intact animal model of Alzheimer’s disease (AD; Furman et al., 2012). It was shown that the bilateral administration of AAV2/5 Gfa2–VIVIT (a synthetic peptide that blocks the calcineurin (CN)/nuclear factor of activated T cells (NFAT) pathway which regulates several components of the activated astrocyte phenotype) into the hippocampus of 7- to 8-month-old APP/PS1 mice, was associated with reduced glial activation, lower amyloid levels, improved synaptic plasticity, and an improved cognitive function at 16–17 months of age. This result represents a proof-of-principle that astrocytes can be considered as significant therapeutic targets not only in AD but also for other neurodegenerative diseases. Because of its specificity, lack of toxicity and capacity for widespread and long-lasting transgene expression, AAV appears to be an ideal vehicle for directing therapeutics to astrocytes.

## CONCLUSION AND PERSPECTIVES

The growing importance of astrocytes in crucial brain functions and also dysfunctions has led to the development of new genetic tools to label and manipulate these glial cells *in vivo.* Thanks to these tools that include targeted transgenesis and viral transduction, considerable advances were made in the understanding of astroglial biology. This first generation of astrocytic viral vectors was instrumental to start depicting their role in specific brain regions of different species. However, a better determination of the numerous functions played by astrocytes during development, in adulthood and disease will require new viral vectors that can further resolve the intimate relationship between neurons and glia in the maturing brain (Molofsky et al., 2012). One important issue relates to the recent but well-accepted notion that astrocytes do not represent a homogenous population of cells. This is, of course, thoroughly demonstrated for neurons (Miller and Gauthier, 2007) but is just starting to be studied for astrocytes in particular because of the lack of reliable markers to follow these different cell populations. The launching of recent initiatives such as the Human Brain Project and ENCODE will increase our knowledge on the functions of astrocytes and may help to refine strategies previously developed to drive transgene expression into specialized astrocytes at different stages of development either in normal or diseased states. A comprehensive mapping of the cell-type-specific expression of miRNAs, the development and *in vivo* assessment of efficient miRT sequences will also permit one to ameliorate the detargeting strategy. Similarly, the identification of the receptors required for the binding of the viral particles to astrocyte subpopulations will represent a major step toward the production of more efficient astrocytic viral vectors. In addition to these strategies which are already used to drive the tropism of viral vectors toward astrocytes, new viral vectors could be developed. Among these emerging viral vectors, baculoviral vectors take advantage of their natural tropism for astrocytes (Boulaire et al., 2009). Their large genome size (140 kb) is suitable for the incorporation of large genes of interest and complex regulatory elements (Wang and Wang, 2006). Clinical observations in patients suffering from neurological pathologies following viral infections suggest that other viruses could have a cerebrotropism (e.g., alphaviruses or arboviruses; Das et al., 2010; Walker et al., 2012). This illustrates the need for multidisciplinary programs that would share the expertise of neurobiologists, virologists, geneticists, and clinicians in order to overcome the limitations of current vectors and discover innovative gene transfer systems. Considering how much more might be discovered about the functions of normal or diseased astrocytes, it is tempting to suggest that we are just at the beginning of the development of astrocentric viral vectors.

## Conflict of Interest Statement

The authors declare that the research was conducted in the absence of any commercial or financial relationships that could be construed as a potential conflict of interest.
